# Fine intercellular connections in development: TNTs, cytonemes, or intercellular bridges?

**DOI:** 10.15698/cst2020.02.212

**Published:** 2020-01-07

**Authors:** Olga Korenkova, Anna Pepe, Chiara Zurzolo

**Affiliations:** 1 Unit of Membrane Traffic and Pathogenesis, Institut Pasteur, 28 rue du Dr Roux, 75015 Paris, France.; 2 Université Paris-Sud, Université Paris-Saclay, 91405 Orsay, France.

**Keywords:** tunneling nanotubes, TNTs, cytonemes, intercellular bridges, intercellular communication

## Abstract

Intercellular communication is a fundamental property of multicellular organisms, necessary for their adequate responses to changing environment. Tunneling nanotubes (TNTs) represent a novel means of intercellular communication being a long cell-to-cell conduit. TNTs are actively formed under a broad range of stresses and are also proposed to exist under physiological conditions. Development is a physiological condition of particular interest, as it requires fine coordination. Here we discuss whether protrusions shown to exist during embryonic development of different species could be TNTs or if they represent other types of cell structure, like cytonemes or intercellular bridges, that are suggested to play an important role in development.

## INTRODUCTION

In 2004, Rustom and colleagues observed ultrafine, short lived, intercellular connections formed between cultured rat pheochromocytoma PC12 cells, human embryonic kidney (HEK) cells and normal rat kidney (NRK) cells [1]. These structures appeared to transfer vesicles between connected cells and were named tunneling nanotubes (TNTs) to distinguish them from other cell protrusions, and for their unique property to provide a seamless continuity between the cytosols of connected cells, as tunnels would do. TNTs were hovering above the substrate and bridging cells over long distances. Strikingly, while TNTs could reach the length of several cell diameters without attaching to a substrate, they didn't appear to contain microtubules, but only actin filaments. Based on their evidence, the authors proposed that the key characteristic of TNTs was that they were able to establish cytoplasmic continuity and this in turn would allow the transfer of membrane vesicles between connected cells [1]. Cells had long been known to form informational networks by contacting neighboring cells as well as by releasing diffusible messengers that spread over long distances, but the possibility that cells could open up to each other (even if only transiently) for a direct long-distance communication was not taken into consideration as it went against the dogma of cells as distinct entities. Indeed, the hypothesis was received with some skepticism from the scientific community [2].

However, during the past 15 years TNT-like structures have been observed in various cell lines in culture, in primary cells and in tissues, and were shown to allow the transfer of different cargoes, ranging from ions to organelles [reviewed in 3, 4–7]. Based on the observations of the transfer of various cargoes, several functions of TNTs have been proposed. Among these, TNTs were shown to be able to electrically couple cells over long distances, enabling Ca^2+^-signaling between connected cells through gap junctions or IP_3_ receptors [3, 8]. This could be consistent with the involvement of TNTs in development, when electrical coupling could help to orchestrate morphogenetic movements, that allow cells to reach their specific niche where differentiation in different tissues or cell types will occur [9, 10]. Specifically, electrical coupling through TNTs could be crucial during early brain development, when neural progenitors have not yet established mature chemical synapses [3–5, 11]. Calcium transfer through TNTs, established between myeloid cells, further suggests the involvement of TNTs in immune cell activation and immune response [8, 12]. TNT involvement in immune response is also supported by the observation of TNT-mediated transfer of the death signal Fas ligand and active caspases, that results in the induction of apoptosis in receiving cells [13].

Last but not least TNTs are also considered to contribute to various pathological conditions. Likewise, the ability of TNTs to form between tumor cells and to connect tumor cells with stromal cells, enabling the transfer of micro-RNAs and mitochondria between connected cells, proposes the involvement of TNTs in cancer progression [7, 14–16]. TNT-like structures were shown to transfer HIV-1 between T-cells and between macrophages [17, 18], which suggests the involvement of TNT-like structures in HIV-1 spreading. Prions and prion-like proteins were also shown to utilize TNTs for their spreading. Specifically, TNTs were shown to transfer PrP^Sc^, alpha-synuclein, tau, beta-amyloid, polyglutamine huntingtin and disrupted-in-schizophrenia 1 (DISC1) aggregates [19–23]. Therefore, it has been proposed that by allowing prion-like protein spreading in the brain, TNTs could have a prominent role in the pathogenesis of different neurodegenerative diseases [22].

One of the major problems in the TNT field is that many different TNT-like structures in different cell types and different conditions have been described [reviewed in 24, 25–28]. As the field still lacks TNT-specific markers, it is still unknown whether there exist one or several types of *bona fide* TNTs (open-ended structures that allow direct communication between cells), and whether there is a structure-function relationship between the different structures. While it is clear that TNTs are different from other closed and shorter cellular protrusions like cilia and substrate attached filopodia [29, 30], it is still problematic to differentiate them from similar structures connecting distant cells like cytonemes and intercellular bridges.

Considering the recent identification of the unique structure of TNTs formed between neuronal cells in culture [31], in this review we will specifically focus on the differences between TNTs and other intercellular structures (e.g. intercellular bridges and cytonemes) both from the structural and functional point of view.

## UNIQUE MORPHOLOGY OF TNTs

The possible TNT involvement in the pathogenesis of diseases such as cancer, AIDS and neurodegenerative diseases has inspired a growing number of studies aimed at TNT characterization. However, TNT investigations face methodological difficulties due to TNT fragility, namely their sensitivity to chemical fixation, mechanical stress and prolonged light excitation [1, 32]. Another problem is the absence of TNT-specific markers, which makes it challenging to identify TNTs and study their functions in tissues. In this context, morphological properties remain the main criteria for TNT identification. The properties commonly used to identify TNTs are: straight, F-actin based bridge-like structure, interconnecting cell pairs; length over several cell diameters and thickness below 1 μm. However, both diameter and length of TNTs vary highly. It was shown that TNT lengths could vary as the connected cells move apart or migrate and therefore the distances between them change. TNT diameter usually ranges between 50 and 700 nm, which also depends on the method used for TNT identification [reviewed in 26, 33]. Moreover, in certain conditions, TNTs were also shown to contain microtubules. As such, apoptotic PC12 cells were shown to form microtubule-containing TNTs, while TNTs of the same PC12 cells formed in normal conditions lack microtubules [34]. TNT-like structures generated by T-cells, that mediate long-distance HIV-1 protein transfer appear to contain microtubules. However, it is not clear whether these are canonical open-ended TNTs or close-ended protrusions (see below) [17]. There is a suggestion to categorize TNTs according to their diameter [35], where «thin» nanotubes display a diameter of up to few hundreds of nanometers and «thick» nanotubes have a diameter of over several hundreds of nanometers. It has also been proposed that «thin» nanotubes could end with gap junctions and allow the exchange of smaller cargo such as molecules below 1.2 kDa, including second messengers and small peptides, while «thick» TNTs can contain microtubules and thus be more stable and be able to mobilize larger cargo such as organelles or viruses [33, 35]. However, these criteria are not stringent enough and seem not to be applicable to all cell types.

**Figure 1:**
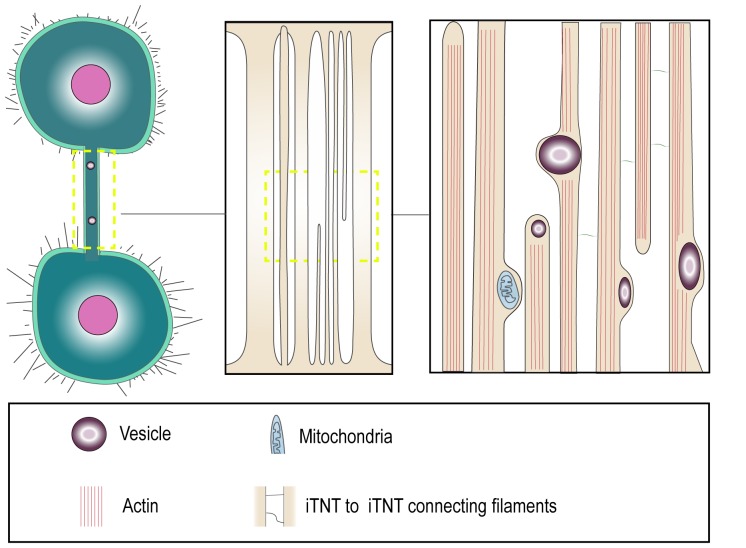
FIGURE 1: “Thick” and “thin” TNT connections. Cryo-electron microscopy shows that TNTs can either be a single thick connection or a bundle of thin individual TNTs (iTNTs). Both open-ended and closed-ended protrusions can be present within a bundle. Each iTNT contain actin bundles, can contain vesicles and mitochondria. Thin and short membrane threads connect several iTNTs, which appear to grow in opposite directions. Adapted from Sartori-Rupp *et al.* [31].

The recent use of correlative light and cryo-electron microscopy has provided several structural details about TNT morphology, that suggest that the terms «thin» and «thick» TNTs should be used with caution [31]. In this study, TNTs were preserved closer to their native status owing to fixation by rapid freezing. This allowed imaging at nanometer resolution by correlative cryo-fluorescent and cryo-electron microscopy and tomography under fully hydrated conditions, which are the best to preserve membrane structures. The findings indicate that the structures that appear to be thicker TNTs by fluorescence microscopy, could in fact be made up of several individual tunneling nanotubes (named iTNTs) **(Figure 1)**. Each iTNT contained actin bundles, which in most cases filled the entire lumen of the tube. The average diameter of iTNTs of about 120 nm and such close-packing of acting bundles did not seem to impede iTNTs to transfer vesicular compartments and even mitochondria within their lumen, as the membrane was often observed to bulge to accommodate the passage of the vesicle **(Figure 1)**. Furthermore, cryo-electron tomography demonstrated the existence of thin short filaments labeled by N-cadherin antibodies, that appeared to connect iTNTs between each other, possibly for holding them in a bundle and conferring higher mechanical stability [31] **(Figure 1)**. In addition to iTNT bundles, single thicker TNTs (600-900 nm in diameter) were also observed. It is important to note that the iTNT bundles and the thicker single TNTs could not be distinguished by fluorescence microscopy as they had similar appearance. These findings suggest that it might be inaccurate to apply diameter-based TNT categorization for TNT identification by fluorescence microscopy, as «thick» TNTs could equally represent bundles of iTNTs which initially have the same diameter as «thin» TNTs.

Moreover, cryo-electron tomography in neuronal cells showed that both thin and thicker iTNTs contained only actin and not microtubules [31], differently from what was proposed as a distinctive feature of thick TNTs in macrophages [35]. Of specific interest, mitochondria were shown to be transported within iTNTs presumably on actin rails, in a tubulin-independent mechanism. This suggests that TNTs do not necessarily need microtubules to allow the transfer of larger cargoes, such as vesicles and organelles [31].

Overall, we propose that such morphological parameters as length, thickness and extension between two cells are insufficient characteristics for TNT identification. On the other hand, as originally proposed by Gerdes and colleagues, the prominent TNT features to be considered for the TNT definition are: 1) establishing cytoplasmic continuity and 2) allowing the transfer of various cargoes including organelles such as lysosomes, endosomes and mitochondria.

Although cytoplasmic continuity has been listed as a fundamental definition criterion of TNTs, it has been challenging to clearly demonstrate it. We specifically addressed the question whether the TNTs we observed in neuronal CAD and SHSY5Y cell lines are in fact connecting the cytosol of two cells. To this aim, we performed Focused Ion Beam Scanning Electron Microscopy (FIB-SEM), which allowed us to image the ends (or contact sites) of TNTs. Interestingly, we were able to identify both open- and close-ended connections such as invaginations at contact zones **(Figure 1)**. We could not discriminate whether the differences observed were the result of the existence of distinct types of TNTs or whether it is due to temporal pre- or post-fusion events. Nonetheless these data represent the first direct confirmation that open-ended TNTs exist and could correspond to the functional TNT structures observed by fluorescent microscopy [31]. Interestingly, we could not observe the presence of gap junction complexes at the end of the thin iTNTs as compared to the thick ones, while they were observed in other cell types [27, 36]. It could be interesting to further investigate TNT structures in the future to understand whether the presence of connexins at the tip of TNTs is cell type specific and/or could be used to define a specific TNT type.

Cryo-electron tomography analysis has also allowed addressing the morphological differences between filopodia and TNTs in neuronal cells. Filopodia in neuronal cells are isolated protrusions with a similar to TNT average diameter of 200 nm. Compared to TNTs, filopodia have a shorter length range, between one and several microns and do not appear to contain vesicles. Interestingly, filopodia also display various actin arrangements compared to TNTs, namely either tight parallel bundles, but of shorter length compared to TNTs, or parallel bundles intermingled with short-branched filaments that were not observed in iTNTs. However, when observing cross-sections of filopodia, actin filaments were shown to be arranged in a hexagonal array similar to that observed in iTNTs [31].

The fact that TNTs display some differences from filopodia in the actin arrangement suggests that TNTs could be formed as distinct structures from the beginning, rather than from filopodia with subsequent fusion of its tip with the recipient cell. Previous findings on TNT-regulating molecules support this hypothesis. Indeed, actin regulatory molecules can have opposite effects on the formation of TNTs and filopodia. Specifically, filopodia inducing CDC42/IRSp53/VASP network decreased the number of TNT-connected cells and down-regulated TNT-mediated vesicle transfer [30]. Similarly, fascin, previously shown to induce dorsal filopodia in mouse neuronal CAD cells [37], failed to promote TNT formation in the same cells [38]. On the contrary, Eps8 (EGF receptor pathway substrate 8), another actin regulator that has been shown to inhibit filopodia formation in neurons, was shown to be a positive regulator of TNT formation and vesicle transfer in CAD cells [30]. Futhermore, our unpublished data shows that Eps8 increases TNT formation and the transfer of α-synuclein in human neuronal SHSY5Y cells, which suggests that it might be a common TNT activator molecule for neuronal cells. However, it should be noted, that TNTs and filopodia can also share the regulators of their formation. Likewise, Myosin X upregulates the formation of both dorsal filopodia and TNTs in CAD neuronal cells [38]. Hence, the possibility that TNTs could arise from dorsal filopodia should not be excluded.

Overall, we propose that morphological parameters as defined by light microscopy are insufficient characteristics for TNT identification, and studies on TNTs should always be able to assess their functionality in transferring molecules or organelles between connected cells. On the other hand, TNTs have a distinct structure that can be identified by electron microscopy. Nonetheless, it should be noted that there exists another type of protrusion that share with TNTs the ability to allow cytoplasmic continuity and cargo transfer. These are intercellular bridges (IBs), known to interconnect germline cells [39, 40]. In the next section we examine the similarities and differences between IBs and TNTs.

## INTERCELLULAR BRIDGES

**Figure 2:**
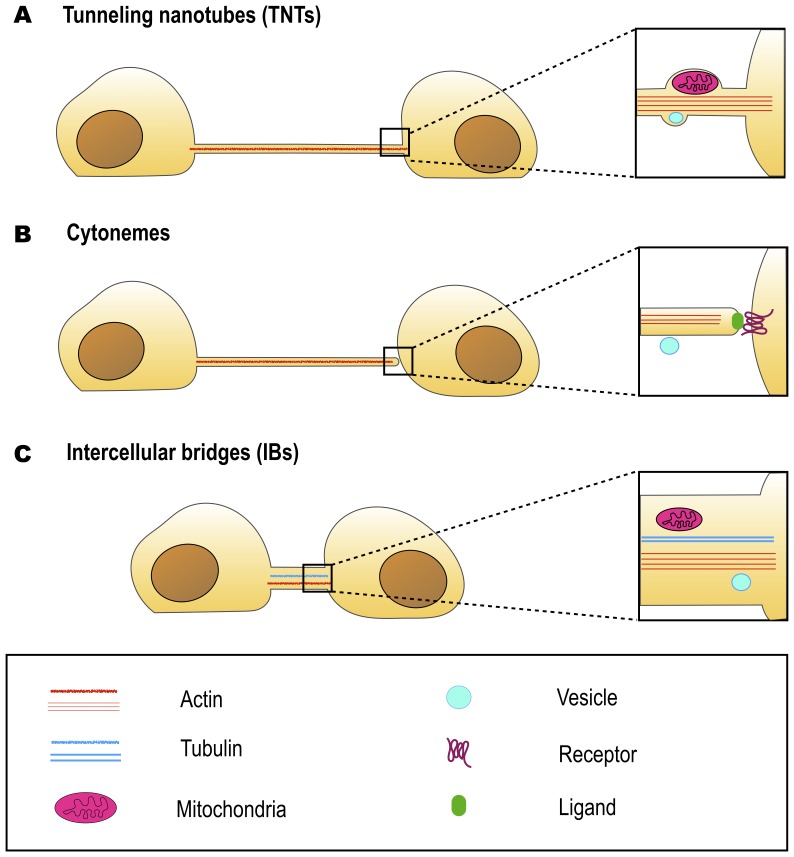
FIGURE 2: Similarities and differences between TNTs, cytonemes and intercellular bridges at optical microscopy level. TNTs **(A)** and cytonemes **(B)** have similar morphologies, they contain actin but not tubulin. However, TNTs are open-ended and allow cytoplasm continuity, while cytonemes are closed-ended, and allow protein-protein interactions. Intercellular bridges **(C)** are open-ended, but are generally shorter and wider than TNTs. Some intercellular bridges were also reported to contain tubulin. All three types of cell structures are able to transfer cargo. However, while cytonemes transfer vesicles on their surface, TNTs and intercellular bridges are able to transfer such organelles as mitochondria in their lumen. As TNT diameter is generally smaller than the diameter of mitochondria, the transfer requires membrane bulging.

Intercellular bridges (IBs) are cytoplasmic bridges that form by incomplete cytokinesis and interconnect cells in syncytia. Stable IBs are found in the female and male germline in different organisms ranging from insects to humans. This evolutionary conservation suggests that this germ cell interconnection is important, and stable intercellular bridges have indeed been shown to be required for fertility [40]. They establish cytoplasmic continuity and are large enough (diameter range of 0.2–10 μm) to allow organelles and/or macromolecules to pass through [41]. Actin and anillin are common components of IBs, while tubulin was only found in male germline IBs **(Figure 2C)** [40]. In many insects, the IBs between female germline cells allow the directional transport of nutrients to promote the growth of one of the cells that will develop into the oocyte, whereas the other cells, after contributing their cytoplasmic contents to the oocyte, retract and die [42]. Such IBs in *Drosophila melanogaster* are called ring canals and grow from a diameter of about 0.5–1.5 μm in the germarium to approximately 10 μm at late stages of oogenesis [42, 43]. As the diameter of the ring canal increases, its length also changes (from 0.22 μm at early stages to 1.87 μm at late stages) [43], however, it remains quite short as compared to TNT length (5-200 μm) **(Table 1)**. Mammalian female germ cells are also connected by IBs, however, their diameter does not exceed 1 μm [40]. Male germline IBs of different species have the same average diameter of 1–1.5 μm, and promote germ cell communication and sharing of cytoplasmic constituents, thereby synchronizing mitotic cell divisions and entry into meiosis [40]. Interestingly, in addition to germline bridges, somatic stable IBs have been described in certain developing invertebrates. The morphology of *Drosophila* somatic IBs differs from that of ring canals. They have a stable diameter of about 0.25 to 1 μm (which is closer to TNT diameter of 0.2 - 0.7 μm), and a length of 0.40 μm (which is very small compared to TNT length of 5-200 μm) **(Table 1)** [44]. It is considered that somatic IBs promote exchange of cytoplasm and maybe organelles, thereby facilitating intercellular communication, synchronization of cell division or differentiation, and coordination of cell behavior during development [40].

**Figure 3:**
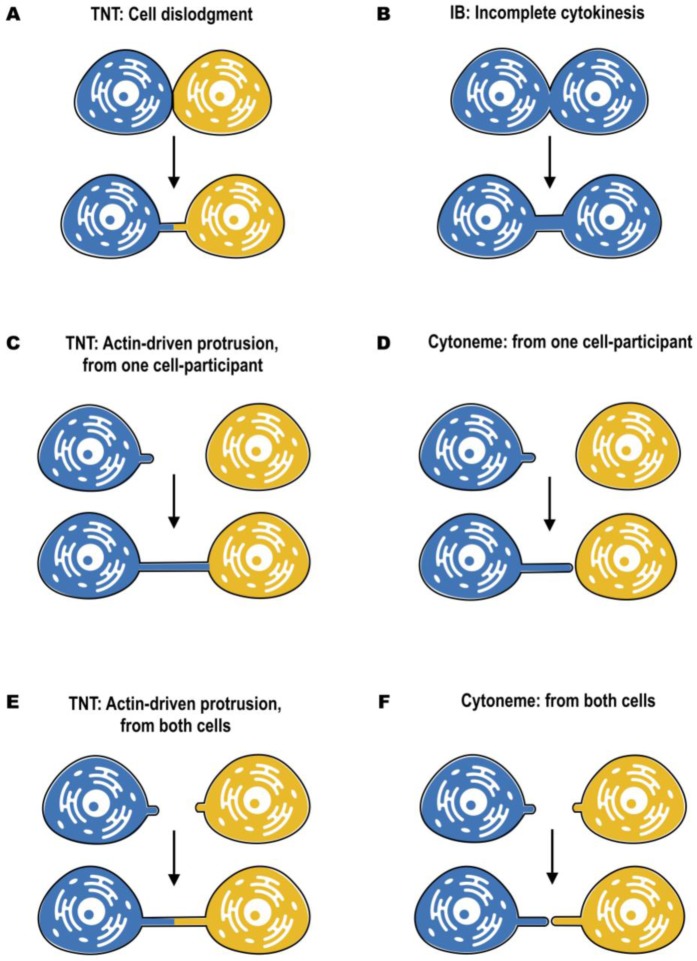
FIGURE 3: Mechanisms of formation of TNTs, cytonemes and intercellular bridges, according to optical live microscopy. TNTs can be formed by cell dislodgment **(A)**, when cells first make contact and then form a nanotube while migrating in the opposite directions. Morphologically it resembles the formation of intercellular bridges **(B)**. However, intercellular bridges are formed only between dividing cells by incomplete cytokinesis, while TNTs can be formed by cells of different origin. Thus, formation of TNTs requires membrane fusion prior to cell dislodgement, while formation of intercellular bridges requires inhibition of abscission after cell division. Another mechanism of TNT formation, called “actin-driven”, takes place when either one **(C)** or both **(E)** of the cells induce the outgrowth of filopodia-like protrusions. Similar mechanism is shown for cytonemes. Cytonemes can be formed from producing to receiving cells and vice versa **(D)**, as well as from both of them **(F)**. However, in case of cytonemes, the outgrowth is finalized by establishing the contact, while TNT formation require subsequent membrane fusion.

In most cases, distinct morphology of IBs makes them easier to distinguish from TNTs **(Figure 2)**. Intercellular bridges generally have shorter and thicker dimensions, interconnecting neighboring cells, while TNTs are generally thin and long, and thus can connect cells over long distances. Nonetheless, a recent study in developing zebrafish gastrula showed that IBs are also able to connect cells over long distances (see below) [45]. In this light, the difference in the mechanisms of formation becomes a more important criterion to distinguish these two structures. While intercellular bridges are formed between dividing cells by incomplete cytokinesis, TNTs are not the result of incomplete cytokinesis and are formed *de novo* between two distinct cells either by growing a filopodia-like protrusion [1] or by dislodgement of two attached cells, leaving TNT as a tether [17] **(Figure 3)**. Thus, while the proposed functions of IBs could overlap with the proposed functions of TNTs during development, they clearly represent distinct structures and, therefore, their purpose should also be different. Specifically, TNTs can also connect cells of different origins. This could happen, for example, in the case of filopodial connections observed during development between ectodermal and mesenchymal cells in sea urchin gastrula [46] or between the cells of inner cell mass and mural trophectoderm cells in mouse blastocyst [47]. More studies will be needed to investigate whether the filopodial connections established in these cases between different cell types are veritable TNTs (see below).

Cytonemes represent another type of cell protrusion morphologically close to TNTs, but having a distinct function **(Table 1)**. Therefore, they might be confused with TNTs if functional characterization is missing. In the next sections we discuss their roles and similarities to TNTs in detail.

**TABLE 1.** Key characteristics of tunneling nanotubes, intercellular bridges and cytonemes.

**Tunneling nanotubes (TNTs)**		**Intercellular bridges (IBs)**		**Cytonemes**
		**Germline IBs**	**Somatic IBs**	
Diameter	50-700 nm	0,5-10 μm	200-1000 nm	100-400 nm
Length	5-200 μm	0,2-2 μm	Average 0,4 μm, reported up to 350 μm	1-700 μm
Composition	Actin	Actin, anillin, δ-tubulin	Actin, anillin	Actin
Formation	Actin-driven protrusion, Cell dislodgement	Incomplete cytokinesis	Incomplete cytokinesis	Actin-driven protrusion
Function	Electrical coupling, organelle transfer, transfer of infectious agents	Germ cell communication, sharing of nutrients, synchronisation of cell division	Coordination of cell division and differentiation during development	Morphogen transfer, morphogen signaling, maintenance of the stem cell niche

## CYTONEMES

Cytonemes were first noted as long cellular extensions that protrude from *Drosophila* wing imaginal disc cells. These protrusions contained actin, but not microtubules, had the length of several cell diameters (up to 700 μm) and the maximum diameter of 200 nm. They were named cytonemes («cell threads») to distinguish them as filopodia of a special type [48]. But the defining characteristic of these protrusions was that, irrespective of cell location in the wing primordium, they oriented uniformly toward the disc midline where the morphogen signaling protein Decapentaplegic (Dpp) is expressed. The presence of such long filopodia that extend between wing disc morphogen-receiving cells and the morphogen-expressing cells suggested an alternative possibility to diffusion-based models of morphogen dispersion [48, 49].

Morphogens are defined as form-giving substances that serve for the generation of positional information in the embryo. According to the model proposed by Lewis Wolpert, morphogens form concentration gradients within a tissue and a specific threshold of each morphogen determines cellular identity [50]. The classic view is that the concentration gradient is formed by simple diffusion. However, a diffusion model appears to be poorly applicable for distinct morphogens, as in the case of Wnt [discussed in detail in 51]. In fact, Wnt ligands are post-translationally lipidated, which generates a poorly soluble, hydrophobic molecule. Numeric simulation suggests that diffusion mechanisms require several tens of hours to establish a stable morphogen gradient and to ensure an appropriate tissue pattern. In contrast, the Wnt morphogenetic gradient operates during a few hours of gastrulation. Moreover, the gastrula stage of embryo development is characterized by highly intensive cellular reorganizations that are likely to impede signaling gradients formed by extracellular diffusion [51]. In this context, cytonemes appear to be a likely mechanism for establishing morphogen gradients not only for Drosophila, but also for vertebrates. Indeed, Wnt cytonemes of the zebrafish embryo were shown to allow fast signaling activation and gradient formation in the recipient cell within minutes [52]. Compared to diffusion-based gradient formation, in the case of cytonemes, Wnt gradients could be dependent on cytoneme length and on the frequency of contact with the recipient cells. Cells that are located closer to the ligand source are contacted more often by Wnt cytonemes than cells further away [51].

In addition to Dpp and Wnt morphogens, cytonemes were shown to contribute to signal transduction in fibroblast growth factor (FGF), epidermal growth factor (EGF), Wingless (Wg), Hedgehog (Hh) and Delta/Notch pathways in Drosophila [reviewed in 29, 49, 51]. Moreover, in addition to Wnt transfer in zebrafish embryo [52, 53], cytoneme-based transfer of Shh (sonic hedgehog) and BMP7 was reported in chicken embryo [54, 55]. Interestingly, it was shown that cells can extend cytonemes that are specific to different morphogens and thus can respond to multiple signals. Specifically, cells of the Drosophila tracheal air sac primordium (ASP) were shown to extend cytonemes containing Dpp-receptor Tkv toward nearby Dpp-producing cells of the wing disc while also directing FGFR-containing cytonemes toward wing disc cells that express FGF. Notably, the examined cytonemes contained only one type of receptor, either Tkv or FGFR, meaning that each cytoneme is specific to only one ligand [56]. Thus, cytonemes could represent a highly specific means of direct intercellular communication during development.

In the context of morphological similarities between TNTs and cytonemes, together with presumably high significance of cytonemes during development, we consider it necessary to clearly discriminate TNTs from cytonemes when studying TNT functions during development. To gain more insight into similarities and differences between cytonemes and TNTs, we will discuss this topic in detail in the next section.

## TNTs AND CYTONEMES: SIMILARITIES AND DIFFERENCES

As previously described, both TNTs and cytonemes are long and thin, F-actin based protrusions and thus morphologically they look very similar. The most prominent feature distinguishing these two types of cell protrusion is that cytonemes are closed-ended, whereas TNTs are open-ended **(Figure 2)**. Subsequently, another key difference lies in their function: while cytonemes allow signal transduction through protein-protein interactions [49], TNTs allow transfer of cargoes through establishing cytoplasm continuity [1, 57].

Given that transfer function is a key feature of TNTs, it is necessary to emphasize that cytonemes also appear to transfer proteins, although this transfer seems to be receptor-dependent [28]. Interestingly, in the pioneering study on Drosophila cytonemes, the authors reported vesicle translocation along their surfaces **(Figure 2B)** [48]. More recently, Shh molecules were shown to transfer in vesicles along the surface of Drosophila cytonemes [58]. Zebrafish cytonemes were also shown to transfer Shh in particles, associated with the external leaflet of the cytoneme membrane [54]. These observations indicate that morphology and the ability to transfer proteins and/or vesicles may be insufficient characteristics to categorize a cell protrusion as a tunneling nanotube, although in the case of TNTs the cargo has been demonstrated to travel inside the tube and not along the membrane surface **(Figure 2)** [31].

As long as both cytonemes and TNTs are F-actin based protrusions, recruitment of the same actin regulators seems likely. Indeed, Myosin X was shown to be associated both with cytonemes [52] and with TNTs [38]. However, in the case of the CDC42/IRSp53 filopodia-inducing pathway [59, 60] it has been shown that while these molecules are able to induce cytoneme formation [52], they appear to inhibit TNT formation [30]. Nonetheless, it should be noted that CDC42 was shown to induce TNTs in non-neuronal cell lines [61]. This suggests that TNT-formation mechanisms could differ in different cells, thus the picture is more complex than anticipated.

For both cytonemes and TNTs, both donor and receiver cells are able to contribute to their formation **(Figure 3)**. Cytonemes were shown to emanate from producing to receiving cells [52], from receiving to producing cells [48], as well as from both of them, resulting in two times elongation of the signaling bridge [51] (**Figure 3D**, **F)**. An elegant mechanism of cytoneme formation and function was recently proposed for Wnt8a-cytonemes in zebrafish [53]. Wnt8a was shown to activate both the PCP (planar cell polarity) pathway by interaction with Ror2 and the β-catenin pathway by interaction with Lrp6. This allows Wnt8a to regulate the formation of cytonemes and its own propagation. In the source cells, Wnt8a binds and activates the PCP pathway. Wnt/PCP influences convergent extension movement and activates GTPase CDC42, which leads to the outgrowth of signaling filopodia. Wnt8a is subsequently loaded onto these cytonemes, and is transported through the tissue to activate the β-catenin pathway in the responding cells, leading to target-gene induction [53].

On the other hand, TNTs can be formed by two different mechanisms. The first one, named actin-driven protrusion mechanism, is based on the ability of either one or both of the cells involved in cell–cell contact to induce the outgrowth of filopodia-like protrusions (**Figure 3C**, **E**). The elongation should then be followed by establishing the contact and membrane fusion [1, 3]. The second mechanism of TNT formation is based on cell dislodgment **(Figure 3A)**, when cells first make contact and then form a nanotube while migrating in the opposite directions [3, 17]. Several pathways involving actin remodeling, Ras GTPases, M-Sec, Rab proteins, and also p53 and EGFR have been described to promote TNT formation in different cellular contexts, suggesting also cell type specificity [30, 38, 62–64]. These mechanisms have been described in details elsewhere [reviewed in 5, 61, 65]. Moreover, a recent study from our laboratory has shown that TNT formation in developing neurons and neuronal cells can be stimulated by the Wnt pathway. However, differently from cytonemes, TNTs are formed following activation of the Wnt downstream Ca^2+^ calmodulin but not of the β–catenin pathway [53, 66]. However, to date the molecular details of the mechanism of TNT formation are largely unclear, notably the molecular machinery mediating cell-to-cell fusion is still obscure.

Interestingly, numerous stimuli that were shown to trigger TNT formation could be defined as «stress». A broad range of stresses, including H_2_O_2_, UV, inflammatory conditions, virus infection, prion aggregation, serum starvation and high-glucose concentration can trigger TNT formation [61, 63, 67–69]. This fact suggests that TNT formation may represent a type of stress response. Once damaged cells connect with healthy cells, cytoplasmic transfer through TNTs could rescue their phenotype and prolong survival [28]. On the other hand, TNTs and TNT-like structures are also frequently observed in stem cells, cancer cells and developing cells [4, 70, 71]. This might indicate that an undifferentiated status could also favor TNT formation. This suggests another possible function of TNTs - as a means of cell fate determination. Overall, these observations support the hypothesis of TNT as a type of intercellular communication: whether there is an external signal, stress or development process, these all are conditions of the changing environment, conditions when communication is actually needed, and when TNTs readily form. More studies will be needed to address this hypothesis and precisely define the molecular signals and components involved in TNT formation both in physiological and pathological conditions.

## INTERCELLULAR CONNECTIONS DURING DEVELOPMENT: TNTs, CYTONEMES OR INTERCELLULAR BRIDGES?

As TNTs are considered to be a means of intercellular communication, one would expect them to form when such communication is needed, namely in the changing environment and in a multicellular organism. In this context, developmental models represent a unique opportunity to investigate native TNTs in physiological conditions.

From the literature discussed above it seems clear that TNTs, cytonemes and IBs are different structures [33, 40]. However, in many cases it turns out to be difficult to definitely categorize the identified protrusion. Likewise, several studies have reported the presence of long thin protrusions during embryonic development of different vertebrate species **(Table 2)** [45–47, 72–75]. Interestingly, protrusions observed in these studies were further referred to as «TNT-like» [4] or «cytoneme-like» [29]. However, none of these studies confirmed the presence of functional TNTs or cytonemes in their models. Some of them rather proposed the presence of IBs of unusual morphology. Here we discuss the studies that describe structures that were not clearly categorized, and compare them with “canonical” TNTs.

**TABLE 2.** Non-classified cell protrusions, described in development.

**Ref.**	**Model**	**Cells that formed protrusions**	**Parameters of described protrusions**						**Proposed biological function of protrusion(s)**	**Possible categorisation of protrusion(s)**
			**Diameter**	**Length**	**Composition**	**TNT characteristics**	**Cytoneme characteristics**	**IB characteristics**		
**[46]**	Sea urchin gastrula	Primary mesenchyme cells	0,2 - 0,4 μm	12 - 80 μm	Actin	Bulges that moved along the protrusions	-	-	Not migration, rather signaling	TNTs
**[47]**	Whole mouse blastocyst culture	Cells of inner cell mass and mural trophectoderm cells	0,2 - 0,4 μm	Up to 35 μm	Actin	Bulges that moved along the protrusions	Presence of EGF and FGF4 receptors on the protrusions	-	Promotion of proliferation signals	Both TNTs and cytonemes
**[72]**	Whole chick embryo explant culture	Cranial neural crest cells	Up to 1 μm	20 - 100 μm	N/A	-	-	Formation associated with cell division	Synchronization of migration	Intercellular bridges and/or TNTs/cytonemes
**[74]**	Whole chick embryo explant culture	Cranial neural crest cells	0,5 - 2 μm	In average 36 μm	N/A	13% of protrusions enabled cytoplasm transfer	87% of protrusions did not enable cytoplasm transfer	The subset of protrusions was formed by dividing cells	Intercellular communication	TNTs, cytonemes and IBs
**[73]**	Whole mouse embryo culture, neural tube closure	Non-neural ectoderm cells	Up to 1 μm	20 - 50 μm	N/A	Subset of the protrusions contained 0,5 μm vesicular structures	Subset of the protrusions had bulbous ends that resided unattached	-	Neural tube closure coordination	Both TNTs and cytonemes
**[45]**	Zebrafish gastrula	Epiblast cells	Up to 1 μm	Up to 350 μm	Actin, tubulin in proximal regions	Membrane continuity	-	Formation associated with cell division	Intercellular communication	Intercellular bridges and/or TNTs
**[75]**	Xenopus early blastula	Blastomeres	200-700 nm	Up to 250 μm	Actin, no tubulin	Transfer of vesicles between cells	-	-	Intercellular communication	TNTs

N/A — not available.

The first description of TNT/cytoneme-like structures in the embryo was in 1995, when Miller and coworkers reported the presence of thin actin-containing filopodia, extending from ectodermal cells, primary mesenchyme cells and secondary mesenchyme cells in sea urchin gastrula [46]. The thin filopodia extended by ectoderm cells were short (5-10 μm) and short-lived, relative to those extended by mesenchyme cells. Primary mesenchyme cells (PMC) formed filopodia with average length of 12 to 20 μm, with distinct filopodia reaching the length of over 80 μm. The diameter was approximately 200-400 nm, although the uniformity in the diameter of thin filopodia was sometimes interrupted by a bulge that travelled towards the cell body. During their migratory phase PMCs were shown to extend few thin filopodia, whereas the greatest abundance of thin filopodia appeared when active PMC migration was over. Moreover, thin filopodia were prevalent at positions in the embryo and at times when earlier studies showed PMCs interacting either with the ectoderm or with secondary mesenchyme cells to transfer patterning or lineage information. Given this fact and that formation of observed filopodia was not associated with migration, the authors suggested a signaling function for these protrusions. Moreover, the authors speculated that given the thin and long morphology of filopodia, diffusion would be too slow for signal transduction and thus an active process, such as electrical stimuli or a second messenger cascade, is more likely to be involved. The authors also proposed that retrograde transport may provide an active means of cell communication, as evidenced by the bulges that were observed moving from filopodial tip to cell body [46]. In other words, the authors proposed the involvement of cytonemes and/or TNTs in cell communication in sea urchin embryo before the terms «cytonemes» and «TNTs» were actually designated.

The study of Salas-Vidal and Lomeli of 2004 represents another study that proposed the presence of TNTs and/or cytonemes during development without actually using these terms [47]. These authors studied the possible means of communication between cells of inner cell mass (ICM) and mural trophectoderm (mTE) cells in a whole mouse blastocyst culture. During growth of the blastocyst, cell proliferation in the trophectoderm (TE) depends on interactions with the ICM and thus becomes progressively restricted to the polar region, which remains close to the ICM. However, some TE cells in the mural region, which lose contact with ICM cells in the course of blastulation, still divide for some time. The authors questioned how these distant cells could receive proliferating signals from the ICM. In fact, they managed to detect long cell projections extending across the blastocoele and linking ICM cells to mTE cells. The projections were straight and long (up to 34.6 μm), the length depending on the degree of blastocoele expansion. These traversing filopodia had the diameter of 200-400 nm at the narrowest point and were shown to contain cytoplasm and actin. The authors were also able to identify bulges that moved along the projection and resembled vesicles that presented retrograde movement with a distinct saltatory and continuous back and forward trajectory. These protrusions might thus represent TNTs, described by Gerdes and collaborators for the first time in the same year [1]. However, the authors also showed signaling, or cytoneme characteristics of these protrusions. In fact, they detected the presence of EGF receptor ErbB3 and FGFR2 receptors on the projections that emanate from TE cells. The presence of these receptors in traversing filopodia is significant considering that their ligands EGF and FGF4 are able to promote proliferation in TE cells and that some mTE cells are known to still divide when they are distant from the ICM. The authors suggested that ICM cells could promote proliferation of mTE cells by EGF and FGF signaling through long traversing filopodia [47]. Overall, this study suggests the existence of protrusions that share the properties of cytonemes and TNTs and raises the question if cytonemes and TNTs are actually distinct types of cell structures.

Also in 2004, Teddy and Kulesa reported the presence of long and thin protrusions in chick embryos while studying neural crest cell migration in whole embryo explant cultures [72]. The cranial neural crest is a highly invasive subpopulation of cells. However, cranial neural crest cells do not invade, but rather sort into and migrate along stereotypical streams to further pattern peripheral structures of the face and neck. Examining neural crest cells within the streams, the authors observed two distinct cell morphologies and named them «bipolar cells» and «hairy cells». «Bipolar» cells had the feature of having few long filopodia that typically aligned in the direction along the cell's trajectory. These filopodia were approximately 1 μm in diameter and had a length of 50-60 μm, but could extend up to 100 μm. On the contrary, «hairy» cells had a large number of filopodia that did not appear to be distributed or aligned in any specific direction and consisted of both long and short lengths, ranging from approximately 20 μm to 100 μm. Interestingly, a typical neural crest cell stream had the feature of «hairy» cells at the front and «bipolar» cells in the middle of the stream. Filopodia formed by neural crest cells in the middle of the stream were shown to make contacts between cells. This contact seemed to result in that a following cell changed the direction of its movement towards a downstream cell [72]. These findings suggest that during migration cells actively form filopodia not only for movement, but also for signaling purpose. Given the fact that cells generally started the migration in the stream in «hairy» morphology, it can be speculated that cells could form numerous protrusions in different directions when seeking for initial information about migration direction. Later on, cells could form fewer protrusions towards the direction of migration to synchronize their migration with other cells of the stream. This study proposes the presence of signaling filopodia during neural crest cells migration in chick embryo, which might represent cytonemes or TNTs. However, the authors noted the association of protrusion formation with cell division. This might mean that these protrusions could rather be intercellular bridges having however more extended morphology than commonly observed.

In a subsequent study by the same group [74], the authors studied communication routes of neural crest cells in chick embryos by following the movement of photoconverted fluorescent molecules. The authors discovered that in a small number of *in vivo* neural crest cell contacts (around 13%), photoconverted fluorescent molecules moved from one neural crest cell into a neighboring neural crest cell through a thin (0.5–2 μm wide) cellular bridge. In contrast, in the majority of cases (around 87%) photoconverted fluorescent molecules traveled to a midpoint in the cellular extensions, identifying the point of contact between the two neighbors. The average length of a cellular bridge was approximately 36.2 μm and the average width was 2.3 μm at the midpoint between cells. In the majority of the observations of cytoplasm transfer between neural crest cells, the transfer speed was higher than random diffusion theoretical value, suggesting an active transfer mechanism [74]. This study allows to speculate that both cytonemes (contacts without cytoplasm transfer) and TNTs (contacts with cytoplasm transfer) could be found in neural crest cells in chick embryo at the same time points. However, observed protrusions were thicker than common cytonemes and TNTs. This might indicate either the fact that cytonemes and TNTs could have more variable morphology than proposed, or that observed structures represent a distinct type of protrusions.

In 2010, Pyrgaki and coworkers used whole embryo culture systems in combination with live imaging of a genetically-encoded reporter to visualize neural tube formation in the developing mouse embryo, with the emphasis on the last step of the process, neural fold fusion [73]. The authors proposed that they had detected both cytoneme-like and TNT-like structures in their model. Cytoneme-like structures were represented by short and long (over 50 μm) flexible cell extensions with bulbous ends that emanated from non-neural ectoderm cells and resided in the gap between the folds. TNT-like structures were described as cellular “bridges” spanning the gap between the two neural folds at the moment when they come close to each other (≈20 μm). These cellular bridges were less than 1 μm in width and contained structures ≈0.5 μm in diameter that were highlighted by the myristoylated Venus reporter, suggesting vesicle nature of these inclusions. The observed bridges were considered not to be IBs, as the observations proposed their *de novo* formation. Indeed, these structures were observed to extend over the physical gap prior to the folds coming together and hence could represent neither the remnants of cell division nor the remnants left by pulling apart of the two folds during a transient separation [73].

In 2011, Caneparo and coworkers detected the presence of IB-like structures linking distant pairs of epiblast cells during the early phases of zebrafish gastrulation [45]. Characterization of these intercellular bridges showed minor variations in diameter (up to 1 μm) and significant variations in length, reaching lengths up to 350 μm with average span of 215 μm. Imaging revealed that actin was present for all of the length, while tubulin was detected only in the proximal region of the IBs. Membrane continuity was confirmed by photoconverting membrane-associated Dendra protein in one cell body and following the motions of the photoconverted pool towards the other cell. By time-lapse imaging of embryos expressing the chromatin marker Histone 2B, it was shown that IBs first appeared between dividing cells at the blastula stage and then persisted throughout gastrulation, morphogenetic movements and subsequent cell mitotic activity. These protrusions were found to be quite common in gastrula; about one of five cells were endowed with IBs. Such a high frequency suggests crucial functions of these cell structures in the embryo, however they were shown to lack any obvious orientation or preferential position in the fate map, bridging cells of both distinct and same embryonic regions [45]. The morphological properties of these protrusions together with membrane continuity resemble TNT features. The lack of any obvious orientation also did not correspond with the key feature of cytonemes. However, the authors also observed that the formation of these protrusions was associated with cell division and thus concluded that they could represent a type of IBs of unusual morphology rather than TNTs.

Last but not least, the work of Danilchik and coworkers of 2013 reported the presence of exceptionally long filopodia-like structures spanning the blastocoel of *Xenopus laevis* early blastula [75]. These structures were organized as arrays of parallel projections that stably connected blastomeres over the distances of up to 250 μm. These connections contained actin, but not tubulin, had diameters ranging from 200 to 700 nm and allowed the transfer of membrane vesicles between cells. They were initially formed between sister-blastomeres and elongated due to blastomere separation driven by blastocoel expansion. These projections persisted for several hours, through multiple rounds of division (i.e. from 32-cell stage up to 256-cell stage). Although described protrusions were formed between dividing blastomeres, the authors were able to distinguish them from IBs due to their morphology and specific arrangement. In fact, the authors also described blastomeres that were still connected by the IB and at the same time projected numerous long filopodia in multiple directions [75]. This observation leads us to the suggestion that different types of protrusions could be formed by the same cell at the same time, and one should not exclude the possibility of TNT and/or cytoneme formation by dividing cells. Consequently, association of protrusion formation with cell division, shown in the works described above [45, 72], does not necessarily mean that these protrusions are not cytonemes or TNTs.

All the studies discussed above have shown the existence of cytoneme-like and TNT-like structures *in vivo* during embryo development. However, none of them confirmed the functionality and hence the identity of the structures observed. Further functional analysis is needed to confirm or confute the presence of TNTs and/or cytonemes in these models and to assess a possible role of TNTs during development.

## CONCLUSIONS

One purpose of this review is to inspire further investigations on the role as well as on functional and structural similarities of TNTs and other protrusions in the developmental models.

Of particular interest is the question whether true TNTs exist *in vivo* during embryonic development, because in contrast to cytonemes, TNT existence in the embryo has not yet been shown. While the functions of cytonemes during development are actively studied, the possible functions of TNTs during development are for the moment only speculated. The main consideration is that TNTs, by electrically coupling the cells of the embryo, could contribute to the coordination of their migratory activity and thus orchestration of morphogenic movements [4, 5]. However, at the same time, similarly to germline intercellular bridges, TNTs could contribute to synchronization of cell divisions and programmed cell death [40]. Indeed, TNTs were shown to transfer death signal Fas ligand and active caspases *in vitro* and thus could be able to induce apoptosis in receiving cells [13]. Another possibility is that TNTs could contribute to cell fate determination. This suggestion is supported by the *in vitro* observation that TNT-like structures formed between mesenchymal stem cells and cardiomyocytes or smooth muscle cells are necessary for stem cell differentiation into cardiomyocytes or smooth muscle cells, respectively [76]. While such functions of TNTs in development have been proposed, the actual functions of TNTs, as well as their existence in the embryo, remain to be investigated. We believe that novel live imaging and cryo-electron microscopy techniques will help to address these questions as well as to assess morphological and functional differences between TNTs and other cell protrusions.

## Abbreviations:

Dpp: – Decapentaplegic,
EGF: – epidermal growth factor,
EPS: – EGF receptor pathway substrate,
FGF: – fibroblast growth factor,
Hh: – hedgehog,
IB: – intercellular bridge,
ICM: – inner cell mass,
iTNT: – individual TNT,
mTE: – mural TE,
PCP: – planar cell polarity,
PMC: – primary mesenchyme cell,
Shh: – sonic Hh,
TE: – trophectoderm,
TNT: – tunneling nanotube.

## References

[1] Rustom A, Saffrich R, Markovic I, Walther P, Gerdes HH (2004). Nanotubular highways for intercellular organelle transport.. Science.

[2] Baker M (2017). Lines of communication.. Nature.

[3] Abounit S, Zurzolo C (2012). Wiring through tunneling nanotubes–from electrical signals to organelle transfer.. J Cell Sci.

[4] Gerdes HH, Rustom A, Wang X (2013). Tunneling nanotubes, an emerging intercellular communication route in development.. Mech Dev.

[5] Sisakhtnezhad S, Khosravi L (2015). Emerging physiological and pathological implications of tunneling nanotubes formation between cells.. Eur J Cell Biol.

[6] Vignais ML, Caicedo A, Brondello JM, Jorgensen C (2017). Cell Connections by Tunneling Nanotubes: Effects of Mitochondrial Trafficking on Target Cell Metabolism, Homeostasis, and Response to Therapy.. Stem Cells Int.

[7] Hekmatshoar Y, Nakhle J, Galloni M, Vignais ML (2018). The role of metabolism and tunneling nanotube-mediated intercellular mitochondria exchange in cancer drug resistance.. Biochem J.

[8] Watkins SC, Salter RD (2005). Functional connectivity between immune cells mediated by tunneling nanotubules.. Immunity.

[9] Tada M, Concha ML (2001). Vertebrate gastrulation: calcium waves orchestrate cell movements.. Curr Biol.

[10] Levin M (2007). Gap junctional communication in morphogenesis.. Prog Biophys Mol Biol.

[11] Wang X, Bukoreshtliev NV, Gerdes HH (2012). Developing neurons form transient nanotubes facilitating electrical coupling and calcium signaling with distant astrocytes.. PLoS One.

[12] Dupont M, Souriant S, Lugo-Villarino G, Maridonneau-Parini I, Verollet C (2018). Tunneling Nanotubes: Intimate Communication between Myeloid Cells.. Front Immunol.

[13] Arkwright PD, Luchetti F, Tour J, Roberts C, Ayub R, Morales AP, Rodriguez JJ, Gilmore A, Canonico B, Papa S, Esposti MD (2010). Fas stimulation of T lymphocytes promotes rapid intercellular exchange of death signals via membrane nanotubes.. Cell Res.

[14] Lou E, Fujisawa S, Morozov A, Barlas A, Romin Y, Dogan Y, Gholami S, Moreira AL, Manova-Todorova K, Moore MA (2012). Tunneling nanotubes provide a unique conduit for intercellular transfer of cellular contents in human malignant pleural mesothelioma.. PLoS One.

[15] Thayanithy V, Dickson EL, Steer C, Subramanian S, Lou E (2014). Tumor-stromal cross talk: direct cell-to-cell transfer of oncogenic microRNAs via tunneling nanotubes.. Transl Res.

[16] Osswald M, Jung E, Wick W, Winkler F (2019). Tunneling nanotube-like structures in brain tumors.. Cancer Rep.

[17] Sowinski S, Jolly C, Berninghausen O, Purbhoo MA, Chauveau A, Kohler K, Oddos S, Eissmann P, Brodsky FM, Hopkins C, Onfelt B, Sattentau Q, Davis DM (2008). Membrane nanotubes physically connect T cells over long distances presenting a novel route for HIV-1 transmission.. Nat Cell Biol.

[18] Hashimoto M, Bhuyan F, Hiyoshi M, Noyori O, Nasser H, Miyazaki M, Saito T, Kondoh Y, Osada H, Kimura S, Hase K, Ohno H, Suzu S (2016). Potential Role of the Formation of Tunneling Nanotubes in HIV-1 Spread in Macrophages.. J Immunol.

[19] Gousset K, Schiff E, Langevin C, Marijanovic Z, Caputo A, Browman DT, Chenouard N, de Chaumont F, Martino A, Enninga J, Olivo-Marin JC, Mannel D, Zurzolo C (2009). Prions hijack tunnelling nanotubes for intercellular spread.. Nat Cell Biol.

[20] Abounit S, Bousset L, Loria F, Zhu S, de Chaumont F, Pieri L, Olivo-Marin JC, Melki R, Zurzolo C (2016). Tunneling nanotubes spread fibrillar alpha-synuclein by intercellular trafficking of lysosomes.. Embo J.

[21] Abounit S, Wu JW, Duff K, Victoria GS, Zurzolo C (2016). Tunneling nanotubes: A possible highway in the spreading of tau and other prion-like proteins in neurodegenerative diseases.. Prion.

[22] Victoria GS, Zurzolo C (2017). The spread of prion-like proteins by lysosomes and tunneling nanotubes: Implications for neurodegenerative diseases.. J Cell Biol.

[23] Zhu S, Abounit S, Korth C, Zurzolo C (2017). Transfer of disrupted-in-schizophrenia 1 aggregates between neuronal-like cells occurs in tunnelling nanotubes and is promoted by dopamine.. Open Biol.

[24] Davis DM, Sowinski S (2008). Membrane nanotubes: dynamic long-distance connections between animal cells.. Nat Rev Mol Cell Biol.

[25] Hurtig J, Chiu DT, Onfelt B (2010). Intercellular nanotubes: insights from imaging studies and beyond.. Wiley Interdiscip Rev Nanomed Nanobiotechnol.

[26] Austefjord MW, Gerdes HH, Wang X (2014). Tunneling nanotubes: Diversity in morphology and structure.. Commun Integr Biol.

[27] Ariazi J, Benowitz A, De Biasi V, Den Boer ML, Cherqui S, Cui H, Douillet N, Eugenin EA, Favre D, Goodman S, Gousset K, Hanein D, Israel DI, Kimura S, Kirkpatrick RB, Kuhn N, Jeong C, Lou E, Mailliard R, Maio S, Okafo G, Osswald M, Pasquier J, Polak R, Pradel G, de Rooij B, Schaeffer P, Skeberdis VA, Smith IF, Tanveer A (2017). Tunneling Nanotubes and Gap Junctions-Their Role in Long-Range Intercellular Communication during Development, Health, and Disease Conditions.. Front Mol Neurosci.

[28] Yamashita YM, Inaba M, Buszczak M (2018). Specialized Intercellular Communications via Cytonemes and Nanotubes.. Annu Rev Cell Dev Biol.

[29] Buszczak M, Inaba M, Yamashita YM (2016). Signaling by Cellular Protrusions: Keeping the Conversation Private.. Trends Cell Biol.

[30] Delage E, Cervantes DC, Penard E, Schmitt C, Syan S, Disanza A, Scita G, Zurzolo C (2016). Differential identity of Filopodia and Tunneling Nanotubes revealed by the opposite functions of actin regulatory complexes.. Sci Rep.

[31] Sartori-Rupp A, Cordero Cervantes D, Pepe A, Delage E, Gousset K, Corroyer-Dulmont S, Schmitt C, Krijnse-Locker J, Zurzolo C (2019). Correlative cryo-electron microscopy reveals the structure of TNTs in neuronal cells.. Nat Commun.

[32] Abounit S, Delage E, Zurzolo C (2015). Identification and Characterization of Tunneling Nanotubes for Intercellular Trafficking.. Curr Protoc Cell Biol.

[33] Mattes B, Scholpp S (2018). Emerging role of contact-mediated cell communication in tissue development and diseases.. Histochem Cell Biol.

[34] Wang X, Gerdes HH (2015). Transfer of mitochondria via tunneling nanotubes rescues apoptotic PC12 cells.. Cell Death Differ.

[35] Onfelt B, Nedvetzki S, Benninger RK, Purbhoo MA, Sowinski S, Hume AN, Seabra MC, Neil MA, French PM, Davis DM (2006). Structurally distinct membrane nanotubes between human macrophages support long-distance vesicular traffic or surfing of bacteria.. J Immunol.

[36] Wang X, Gerdes HH (2012). Long-distance electrical coupling via tunneling nanotubes.. Biochimica et biophysica acta.

[37] Bohil AB, Robertson BW, Cheney RE (2006). Myosin-X is a molecular motor that functions in filopodia formation.. Proc Natl Acad Sci USA.

[38] Gousset K, Marzo L, Commere PH, Zurzolo C (2013). Myo10 is a key regulator of TNT formation in neuronal cells.. J Cell Sci.

[39] Greenbaum MP, Iwamori T, Buchold GM, Matzuk MM (2011). Germ cell intercellular bridges.. Cold Spring Harb Perspect Biol.

[40] Haglund K, Nezis IP, Stenmark H (2011). Structure and functions of stable intercellular bridges formed by incomplete cytokinesis during development.. Commun Integr Biol.

[41] Robinson DN, Cooley L (1996). Stable intercellular bridges in development: the cytoskeleton lining the tunnel.. Trends Cell Biol.

[42] Robinson DN, Cooley L (1997). Genetic analysis of the actin cytoskeleton in the Drosophila ovary.. Annu Rev Cell Dev Biol.

[43] Tilney LG, Tilney MS, Guild GM (1996). Formation of actin filament bundles in the ring canals of developing Drosophila follicles.. J Cell Biol.

[44] Woodruff RI, Tilney LG (1998). Intercellular bridges between epithelial cells in the Drosophila ovarian follicle: a possible aid to localized signaling.. Dev Biol.

[45] Caneparo L, Pantazis P, Dempsey W, Fraser SE (2011). Intercellular bridges in vertebrate gastrulation.. PLoS One.

[46] Miller J, Fraser SE, McClay D (1995). Dynamics of thin filopodia during sea urchin gastrulation.. Development.

[47] Salas-Vidal E, Lomeli H (2004). Imaging filopodia dynamics in the mouse blastocyst.. Dev Biol.

[48] Ramirez-Weber FA, Kornberg TB (1999). Cytonemes: cellular processes that project to the principal signaling center in Drosophila imaginal discs.. Cell.

[49] Kornberg TB, Roy S (2014). Cytonemes as specialized signaling filopodia.. Development.

[50] Wolpert L (1969). Positional information and the spatial pattern of cellular differentiation.. J Theor Biol.

[51] Stanganello E, Scholpp S (2016). Role of cytonemes in Wnt transport.. J Cell Sci.

[52] Stanganello E, Hagemann AI, Mattes B, Sinner C, Meyen D, Weber S, Schug A, Raz E, Scholpp S (2015). Filopodia-based Wnt transport during vertebrate tissue patterning.. Nat Commun.

[53] Mattes B, Dang Y, Greicius G, Kaufmann LT, Prunsche B, Rosenbauer J, Stegmaier J, Mikut R, Ozbek S, Nienhaus GU, Schug A, Virshup DM, Scholpp S (2018). Wnt/PCP controls spreading of Wnt/beta-catenin signals by cytonemes in vertebrates.. Elife.

[54] Sanders TA, Llagostera E, Barna M (2013). Specialized filopodia direct long-range transport of SHH during vertebrate tissue patterning.. Nature.

[55] Schlueter J, Mikawa T (2018). Body Cavity Development Is Guided by Morphogen Transfer between Germ Layers.. Cell Rep.

[56] Roy S, Hsiung F, Kornberg TB (2011). Specificity of Drosophila cytonemes for distinct signaling pathways.. Science.

[57] Gerdes HH, Carvalho RN (2008). Intercellular transfer mediated by tunneling nanotubes.. Curr Opin Cell Biol.

[58] Gradilla AC, Gonzalez E, Seijo I, Andres G, Bischoff M, Gonzalez-Mendez L, Sanchez V, Callejo A, Ibanez C, Guerra M, Ortigao-Farias JR, Sutherland JD, Gonzalez M, Barrio R, Falcon-Perez JM, Guerrero I (2014). Exosomes as Hedgehog carriers in cytoneme-mediated transport and secretion.. Nat Commun.

[59] Disanza A, Mantoani S, Hertzog M, Gerboth S, Frittoli E, Steffen A, Berhoerster K, Kreienkamp H-J, Milanesi F, Fiore PPD, Ciliberto A, Stradal TEB, Scita G (2006). Regulation of cell shape by Cdc42 is mediated by the synergic actin-bundling activity of the Eps8–IRSp53 complex.. Nat Cell Biol.

[60] Disanza A, Bisi S, Winterhoff M, Milanesi F, Ushakov DS, Kast D, Marighetti P, Romet-Lemonne G, Muller HM, Nickel W, Linkner J, Waterschoot D, Ampe C, Cortellino S, Palamidessi A, Dominguez R, Carlier MF, Faix J, Scita G (2013). CDC42 switches IRSp53 from inhibition of actin growth to elongation by clustering of VASP.. Embo J.

[61] Kimura S, Hase K, Ohno H (2013). The molecular basis of induction and formation of tunneling nanotubes.. Cell Tissue Res.

[62] Hase K, Kimura S, Takatsu H, Ohmae M, Kawano S, Kitamura H, Ito M, Watarai H, Hazelett CC, Yeaman C, Ohno H (2009). M-Sec promotes membrane nanotube formation by interacting with Ral and the exocyst complex.. Nat Cell Biol.

[63] Wang Y, Cui J, Sun X, Zhang Y (2011). Tunneling-nanotube development in astrocytes depends on p53 activation.. Cell Death Differ.

[64] Zhu S, Bhat S, Syan S, Kuchitsu Y, Fukuda M, Zurzolo C (2018). Rab11a-Rab8a cascade regulates the formation of tunneling nanotubes through vesicle recycling.. J Cell Sci.

[65] Marzo L, Gousset K, Zurzolo C (2012). Multifaceted roles of tunneling nanotubes in intercellular communication.. Front Physiol.

[66] Vargas JY, Loria F, Wu JW, Cordova G, Nonaka T, Bellow S, Syan S, Hasegawa M, van Woerden GM, Trollet C, Zurzolo C (2019). The Wnt/Ca2+ pathway is involved in interneuronal communication mediated by tunneling nanotubes.. Embo J.

[67] Zhu D, Tan KS, Zhang X, Sun AY, Sun GY, Lee JC (2005). Hydrogen peroxide alters membrane and cytoskeleton properties and increases intercellular connections in astrocytes.. J Cell Sci.

[68] Aggarwal A, Iemma TL, Shih I, Newsome TP, McAllery S, Cunningham AL, Turville SG (2012). Mobilization of HIV spread by diaphanous 2 dependent filopodia in infected dendritic cells.. PLoS Pathog.

[69] Zhu S, Victoria GS, Marzo L, Ghosh R, Zurzolo C (2015). Prion aggregates transfer through tunneling nanotubes in endocytic vesicles.. Prion.

[70] Lou E, Gholami S, Romin Y, Thayanithy V, Fujisawa S, Desir S, Steer CJ, Subramanian S, Fong Y, Manova-Todorova K, Moore MAS (2017). Imaging Tunneling Membrane Tubes Elucidates Cell Communication in Tumors.. Trends Cancer.

[71] Murray LMA, Krasnodembskaya AD (2018). Concise Review: Intercellular Communication Via Organelle Transfer in the Biology and Therapeutic Applications of Stem Cells.. Stem Cells.

[72] Teddy JM, Kulesa PM (2004). In vivo evidence for short- and long-range cell communication in cranial neural crest cells.. Development.

[73] Pyrgaki C, Trainor P, Hadjantonakis AK, Niswander L (2010). Dynamic imaging of mammalian neural tube closure.. Dev Biol.

[74] McKinney MC, Stark DA, Teddy J, Kulesa PM (2011). Neural crest cell communication involves an exchange of cytoplasmic material through cellular bridges revealed by photoconversion of KikGR.. Dev Dyn.

[75] Danilchik M, Williams M, Brown E (2013). Blastocoel-spanning filopodia in cleavage-stage Xenopus laevis: Potential roles in morphogen distribution and detection.. Dev Biol.

[76] Wang T, Xu Z, Jiang W, Ma A (2006). Cell-to-cell contact induces mesenchymal stem cell to differentiate into cardiomyocyte and smooth muscle cell.. Int J Cardiol.

